# *Salmonella* Gallinarum in Small-Scale Commercial Layer Flocks: Occurrence, Molecular Diversity and Antibiogram

**DOI:** 10.3390/vetsci8050071

**Published:** 2021-04-23

**Authors:** A. K. M. Ziaul Haque, Mir Rowshan Akter, SK Shaheenur Islam, Jahangir Alam, Sucharit Basu Neogi, Shinji Yamasaki, S. M. Lutful Kabir

**Affiliations:** 1 Department of Microbiology, Hajee Mohammad Danesh Science and Technology University, Dinajpur 5200, Bangladesh; vetzia.2004.bd@gmail.com (A.K.M.Z.H.); akter.rowshan@gmail.com (M.R.A.); 2 Department of Microbiology and Hygiene, Bangladesh Agricultural University, Mymensingh 2202, Bangladesh; s_islam73@live.com; 3 Animal Biotechnology Division, National Institute of Biotechnology, Ganakbari, Ashulia, Savar, Dhaka 1349, Bangladesh; alamjahan2003@yahoo.com; 4 Graduate School of Life and Environmental Sciences, Osaka Prefecture University, Osaka 598-8531, Japan; sbneogi@yahoo.com (S.B.N.); shinji@vet.osakafu-u.ac.jp (S.Y.)

**Keywords:** *Salmonella* Gallinarum, *inv*A and *spv*C genes, layer flocks, occurrence, multidrug resistance

## Abstract

*Salmonella* Gallinarum is one of the most important bacterial pathogens associated with diminished egg production in poultry. The aim of this study was to understand the occurrence, molecular traits and antimicrobial resistance patterns of *Salmonella* Gallinarum strains isolated from small-scale commercial layer flocks with low level biosecurity standards in Bangladesh. A total of 765 samples, including cloacal swabs (535), visceral organs (50), and droppings (180), were collected from chickens of 12 layer flocks in 11 districts. *Salmonella* Gallinarum was isolated and characterized through culture-based method, followed by biochemical tests, sero-grouping, PCR assays, sequencing, and antibiogram. The identity of biochemically detected isolates of *Salmonella* Gallinarum was confirmed via genus-specific 16S rRNA gene based PCR, followed by *inv*A and *spv*C genes based PCR assays. Occurrence of *Salmonella* Gallinarum was detected in overall 25.75% (197/765) samples, with a significantly (*p* < 0.05) higher incidence in visceral organs (42%) in comparison to cloacal swab (24%) and droppings (26%). Sequencing and subsequent phylogenetic analysis of *inv*A and *spv*C genes in representative strains of *Salmonella* Gallinarum revealed a close genetic lineage, with a sequence similarity of 98.05–99.21% and 97.51–99.45%, respectively, to previously published sequences of the corresponding genes from the same serogroup strains. Remarkably, 66.5% (131/197) of the isolated strains of *Salmonella* Gallinarum were found to be resistant to 3 to 6 antimicrobial agents, and interpreted as multidrug resistant (MDR). The findings of this study underscore an inherent need of appropriate control measures to curb the widespread incidence of MDR *Salmonella* Gallinarum in small-scale commercial layer flocks, thereby, facilitating enhanced egg production and further support to the food security and safety in low resource settings.

## 1. Introduction

Eggs and meat from poultry are indispensable protein sources in peoples meals in Bangladesh [1]. However, the advancement in the poultry production is often interrupted by the overwhelming occurrence of infectious diseases in low resource settings of developing countries such as Bangladesh. Occurrence of these diseases, which incur a colossal loss due to less production of a quality product and also from the treatment cost, are attributable to noncompliance of good agriculture practices (GAP), low biosecurity, and inadequate hygienic measures in poultry farms [2,3]. Amongst the infectious pathogenic microbes, multidrug-resistant (MDR) *Salmonella* spp. has been regarded as a significant problem to the poultry sector in Bangladesh [4]. *Salmonella* species are rod shaped Gram-negative bacteria, which are predominantly classified as *Salmonella enterica* and *Salmonella bongori*. Salmonella *enterica* subsp. *enterica* comprises more than 1400 serovars, of which approximately 10% are reported from chicken [5,6]. *Salmonella enterica* subsp. *enterica* serovars Gallinarum and Pullorum, predominantly adapted in avian fauna, are closely related serovars, and indistinguishable by culture-based serotyping. However, stringent molecular assays such as enzyme based ribotyping and multiplex PCR method along with biochemical tests can distinguish these closely related serovars of *S. enterica* subsp. *enterica* [7,8]. Among the predominant infectious diseases in the poultry sector, Fowl Typhoid (FT) is a septicemic disease caused by *Salmonella* Gallinarum that mainly affects mature poultry flocks, particularly during the laying period. The disease is clinically manifested by reduced fertility, egg production, and hatchability, leading to increased mortality and considerable financial loss in poultry industries [9].

A variety of virulence factors have been reported for the pathogenic strains of *Salmonella* spp. Occurrence of *inv* genes, which aid the bacterial invasion of host cell, has been observed to be widespread, encompassing ca. 2000 serovars of *Salmonella* spp. [10]. This invasion gene, *invA*, is found to be genetically very similar among the closely related serovars of *Salmonella*, but remains highly conserved in the genome of *Salmonella* Gallinarum, and may be absent in *Salmonella* isolates under the serotypes: Enteritidis, Anatum, and Amsterdam [11,12]. Presence of virulence plasmid, containing pathogenic genes, including *spv*, is considered as an important trait for many pathogenic *Salmonella* serovars, viz., Typhimurium, Choleraesuis, Dublin, Enteritidis, and Gallinarum-Pullorum. The *spv* gene is claimed to facilitate the growth of *Salmonella* in the host environment by interacting with the host immune system [13,14,15]. Considering the above perspectives, PCR assays detecting the invasion gene and plasmid virulence gene might be beneficial for the rapid screening of *Salmonella* species, including *Salmonella* Gallinarum [11]. Therefore, multiplex PCR assays targeting *invA* and s*pvC* genes have been developed and evaluated for the confirmation of *Salmonella* serovars [14,15,16,17]. At present, PCR-based confirmation of *Salmonella* Gallinarum targeting *inv*A and *spv*C genes, using selective primers, S139-S141 [18], and SPV1-SPV2 [13], respectively, is considered to be highly efficient and suitable among the molecular detection tools [19].

In the recent decades, the escalating incidences of antimicrobial resistance (AMR) in pathogenic microbes, including *Salmonella* spp., has been linked to the indiscriminate use of antimicrobial drugs in poultry production [20]. Despite having an enormous potentiality of income generation activities to support the livelihood of millions of people, the recurrent infection of *Salmonella* spp., particularly *Salmonella* Gallinarum, in laying flocks has become a nascent threat for the poultry industry in Bangladesh [21]. A high incidence (53.5%) of *Salmonella* infections in adult layer chickens has been reported for the low resource settings in this country [22]. Moreover, a number of investigations have confirmed wide-scale occurrence of antimicrobial resistance in *Salmonella* spp., with variable incidence, between 20% and 100%, in poultry animals and associated environmental samples in Bangladesh [1,23,24,25,26]. Among different types of salmonellosis in poultry, *Salmonella* Gallinarum can cause more than 70% infection in layer chicken [27]. The economic benefit of poultry farmers is frequently disrupted due to a colossal loss in egg production, which can be attributable to MDR infection of *Salmonella* spp., especially *Salmonella* Gallinarum [10,28,29,30]. Notably, according to a previous report, majority (>80%) of the poultry-originated isolates of *Salmonella* spp. were resistant to commonly used antimicrobials, namely, amoxicillin, doxycycline, kanamycin, gentamicin, and tetracycline [31]. The rampant use of antimicrobials in poultry production, facilitating the emergence of multi-drug resistant (MDR) pathogens, is considered as a major concern to ‘One health’, a framework that holistically considers the health of animals, humans, and the environment while integrating the multifaceted drivers and consequences of infectious diseases [23].

Although a number of studies have revealed a widespread occurrence of *Salmonella* infection in poultry farms [24,26,32,33] little is known on the molecular traits and MDR patterns of *Salmonella* Gallinarum strains circulating among the layer flocks in Bangladesh. Without detailed information on the prevalence, and virulence traits, including AMR patterns of important pathogens, it is difficult to formulate intervention strategies to reduce disease burden in poultry production. Therefore, this surveillance study was conducted involving small-scale commercial poultry farms at different districts in five administrative divisions of Bangladesh to understand: (1) the occurrence, (2) molecular traits, and (3) diversity in antimicrobial resistance patterns of *Salmonella* Gallinarum in layer chicken. With respect to the development efforts aiming to enrich food security, and a more sustainable livelihood of small-scale poultry farmers, the findings of this study are considerably valuable to better understand the risks of infectious pathogens and formulating evidence-based control strategies to reduce disease burden in the poultry sector in Bangladesh and elsewhere.

## 2. Materials and Methods

### 2.1. Study Design and Location

**Figure 1:**
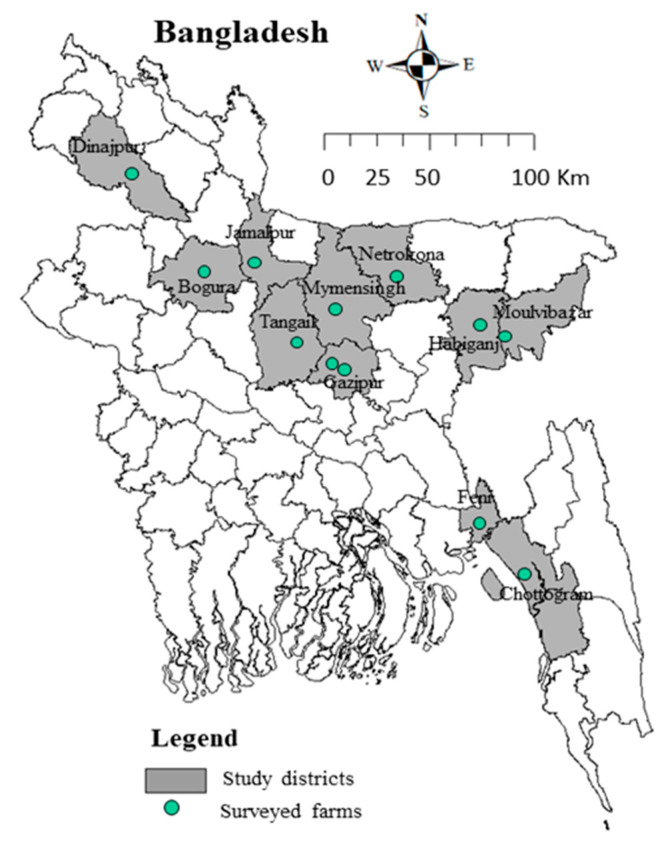
Location of 12 layer flocks of 11 districts (five administrative divisions) of Bangladesh were taken under this survey that involved three from Dhaka, three from Mymensingh, two from Rangpur, two from Sylhet, and two from Chattogram division.

The study was conducted in 11 districts from five administrative divisions (Dhaka, Mymensingh, Rangpur, Sylhet, and Chattogram) of Bangladesh during March to September, 2020. A total of 12 small scale layer farms including three from the Dhaka division (two from Gazipur and one from Tangail districts), three from Mymensingh division (one each from Mymensingh, Jamalpur, and Netrokona districts), two from Rangpur division (one each from of Dinajpur and Bogura districts), two from Sylhet division (one each from Habiganj and Moulvibazar districts), and two from Chattogram division (one each from Chattogram and Feni districts) were enrolled (Figure 1). The farms represented the typical scenario of low-biosecurity standard, and were selected based on the following criteria: flock age of a minimum of 20 weeks, flock size >1000 birds, and lacking any vaccination measure against *Salmonella* Gallinarum. Details of the surveyed farms, including the type, location, and number of collected samples, are presented in Supplementary Table S1.

### 2.2. Sample Collection from the Layer Farms

The samples comprised of cloacal swabs (*n* = 535, 70.39%), poultry drooping (*n* = 180, 23.52%); and visceral organs (*n* = 50, 6.35%), which represented the composite samples of liver, spleen, ovary, and ovarian follicle from the dead layer birds. All samples were collected in sterile plastic containers, properly labeled and transferred in an insulated foam box maintaining cool chain at 4–6 °C to the Department of Microbiology and Hygiene laboratory of Bangladesh Agricultural University and processed within 24 h. During each sampling, information on different parameters as shown in the sample collection checklist (Supplementary Table S2) were obtained.

### 2.3. Isolation and Identification via Culture and Biochemical Tests

Isolation of *Salmonella* Gallinarum was done based on selective enrichment of the samples (cloacal swab, visceral organ, and droppings) in Rappaport Vassiliadis Soya Broth (RVS Broth) (HiMedia, Mumbai, India) following the protocol described earlier [34] with some modifications. A loopful of enriched broth was streaked on xylose lysine deoxycholate (XLD) agar media (HiMedia, Mumbai, India) and incubated at 43 ± 0.2 °C for 24 ± 2 h. Representative colonies of *Salmonella* cells grown on selective XLD agar media were separated individually by subculture on the same media. Thus, the obtained pure culture of each of the selected isolates were subjected to biochemical tests viz., sugar fermentation test, indole, and MR-VP tests [35]; and additionally, motility test was done using the hanging drop slide technique [36] to ascertain their identity as *Salmonella* spp.

### 2.4. Serogrouping of Salmonella Isolates

The isolates of *Salmonella* spp. obtained from the poultry samples were subjected to sero-grouping by rapid serum plate agglutination test (RSPAT) using commercially available *Salmonella* agglutinating antisera (S & A Reagents Lab Ltd., Bangkok, Thailand), and following the standard method described earlier [37].

### 2.5. Molecular Detection

Selected isolates of *Salmonella* spp. were subjected to detection by culture-based methods, and further confirmed by molecular tools, including PCR assays and sequencing. Culture lysate and DNA template of *S*. Gallinarum isolates were prepared using the standard procedure, as described previously [38]. A previously established 16S rRNA gene-based PCR, using primers and conditions shown in Table 1, was performed for confirmation of the genus *Salmonella* [39]. A reaction mixture of 25 μL volume, including 12.5 μL 2Χ master mixture, 2 μL genomic DNA, 1 μL each primer, and 8.5-μL nuclease-free water (Thermo Fisher Scientific, Waltham, MA, USA) was prepared and PCR amplification was carried out using a thermocycler (Astec, Fukuoka, Japan) as per the manufactures’ protocol. The PCR reaction comprised of an initial denaturation step at 94 °C for 5 min; followed by 30 cycles of DNA amplification, each including denaturation at 94 °C for 30 s, annealing at 50 °C for 30 s, and extension at 72 °C for 30 s; followed by a final extension step at 72 °C for 5 min. The PCR products were subjected to gel (2% agarose) electrophoresis (Invitrogen, Carlsbad, CA, USA), followed by staining of the gel with ethidium bromide (0.5 μg/mL) and de-staining in distilled water, each for 10 min.; and finally, the PCR amplicons in the gel were visualized under UV light, and images were captured using a gel documentation system (Biometra, Göttingen, Germany). Species-specific PCR assays, targeting *inv*A and *spv*C genes, were employed to confirm the identity of *Salmonella* spp. isolates [13,18]. Details of the primers used in the PCR assays are listed in Table 1. Initially, a reaction mixture (25 μL) was prepared with template DNA, × 5 PremixTaq™ cradle, deoxynucleotide triphosphates (10 mM each), 25 mM MgCl2, 10 μM of each primer with Premix Taq™ DNA polymerase (Takara Bio Inc., Shiga, Japan), and nuclease-free water in volumes of 3 μL, 5 μL, 0.5 μL, 1.6 μL, 1 μL, 0.3 μL, and 12.6 μL, respectively. PCR amplification, agarose gel electrophoresis, and visualization of the PCR products were done following the standard protocol, as described previously.

### 2.6. Sequencing and Phylogenetic Tree Construction

PCR products of representative isolates, namely BAUSG3 and BAUSG6, were purified using the GeneJET Genomic DNA Purification Kit (Thermo Scientific™, Thermo Fisher Scientific, Waltham, MA, USA); and then sequenced using specific primers partially amplifying the *inv*A and *spv*C genes (Table 1) in a Genetic Analyzer 3130 (Applied Biosystems™, Thermo Fisher Scientific, Waltham, MA, USA) as per manufacturer’s instructions. After initial quality check-up, trimming and editing, as required, the sequenced genes were uploaded in the GenBank. The ClustalW algorithm of the Molecular Evolutionary Genetics Analysis (MEGA) software (version 4.1.0) was applied to align the different sequences of the specific genes, including those obtained in this study and others selected through homology search using BLAST tool (www.ncbi.nlm.nih.gov/BLAST, accessed on 4 December 2020). Afterwards, a phylogenetic tree was constructed following the neighbour-joining (NJ) method [38]. The bootstrap test (100 replicates) was used to evaluate the percentage of replicate trees with related taxa assembled and that appeared as branches, identically [39]. The sequence identity was validated by comparison with the published sequences of the target genes available in the GenBank database (https://www.ncbi.nlm.nih.gov/, accessed on 10 January 2021).

### 2.7. Antimicrobial Susceptibility Screening

Antimicrobial susceptibility patterns of all isolates were determined according to the disc diffusion method [41]. A total of 13 antimicrobials, representing all the major classes, were used for this purpose. All antimicrobial discs were obtained from Oxoid, UK and applied at standard doses: Ciprofloxacin (5 μg), Neomycin (30 μg), Norfloxacin (10 μg), Levofloxacin (5 μg), Enrofloxacin (5 μg), Amoxycillin (10 μg), Amikacin (30 μg), Doxycycline (30 μg), Gentamicin (10 μg), Sulfamethoxazole (25 μg), Azithromycin (15 μg), Tetracycline (30 μg), and Fosfomycin (50 μg). The zones of growth inhibition were compared and interpreted as susceptible (S), intermediate resistant (I), or resistant (R) to the corresponding antimicrobials according to the Clinical and Laboratory Standards Institute [42]. *Salmonella* isolates that demonstrated resistant traits against three or more antimicrobial classes were considered as MDR [43]. *Escherichia coli* ATCC 25922 was used as a quality control organism. All interpretations were corroborated by conducting at least two replicates of the antimicrobial susceptibility testing by this assay.

### 2.8. Data Management and Statistical Analysis

All data obtained in this study were recorded in Excel spreadsheets and analyzed for descriptive statistics (frequency, proportion and 95% Confidence Interval [CI]). To estimate the variations in occurrence and antimicrobial resistance pattern of *Salmonella* Gallinarum, SPSS software (version 22.0, IBM Corp., Armonk, NY, USA) was used. Chi-squared test was performed where applicable to determine the level of significance of difference or association. A *p* value of <0.05 was considered statistically significant.

## 3. Results

### 3.1. Occurrence of Salmonella spp. and Salmonella Gallinarum

#### 3.1.1. Sample Level Occurrence

Among the collected samples (*N* = 765), 28% (*n* = 214) were confirmed as positive for *Salmonella* spp. by conventional culture-based method. Among these isolates (*n* = 214) of *Salmonella*, 199 were confirmed to be *Salmonella* Gallinarum through selective biochemical tests, including carbohydrate fermentation, indole production, methyl red test, Voges-Proskauer reaction, and motility test. Isolates of *Salmonella* Gallinarum produced positive results in fermentation of glucose, maltose, and dulcitol, all without any gas production, which are typical biochmemical traits of this serovar, and useful to differentiate from closely related serovars (Table 2 and Table 3).

The 16S rRNA gene-based PCR confirmed the genus identity of 205 of 214 isolates, biochemically determined as *Salmonella* spp. Results of this genus-specific PCR showed that, overall, an estimated 26.80% (205 of 765) samples were contaminated with *Salmonella* spp., since the representative isolates from the positive samples generated the expected amplicon size of 574 bp (Table 4). However, invA and spvC-gene based PCR assays confirmed that approximately 25.80% (*n* = 197) samples/isolates were positive for *Salmonella* Gallinarum. The identity of these genes was further validated via sequencing, while phylogenetic analysis revealing their closest affinity to corresponding gene sequences reported previously from *Salmonella* Gallinarum strains. Observed variations in the occurrence of *Salmonella* spp. in different kinds of samples was found to be statistically significant (Table 2 and Table 4). Among the four kind of samples, a distinctive higher (*p* < 0.025) occurrence of *Salmonella* spp., including *Salmonella* Gallinarum (mean incidence rate 44% and 42%, respectively), was observed for the tested visceral organs in comparison to cloacal swab and dropping samples (mean incidence rate ca. 25 to 27% and 24 to 26%, respectively).

Of 205 isolates of *Salmonella* spp., when subjected to serogrouping using fur commercially available types of antisera in rapid serum plate agglutination test (RSPAT), the *Salmonella* spp. was classified into three serogroups, viz, with exclusive dominance of Group D (*n* = 199, 97%), and minor presence of Group B (*n* = 4, 2%) and Group C (*n* = 2, 1%) strains (Table 5).

#### 3.1.2. Farm and Division Level Occurrence

**Figure 2:**
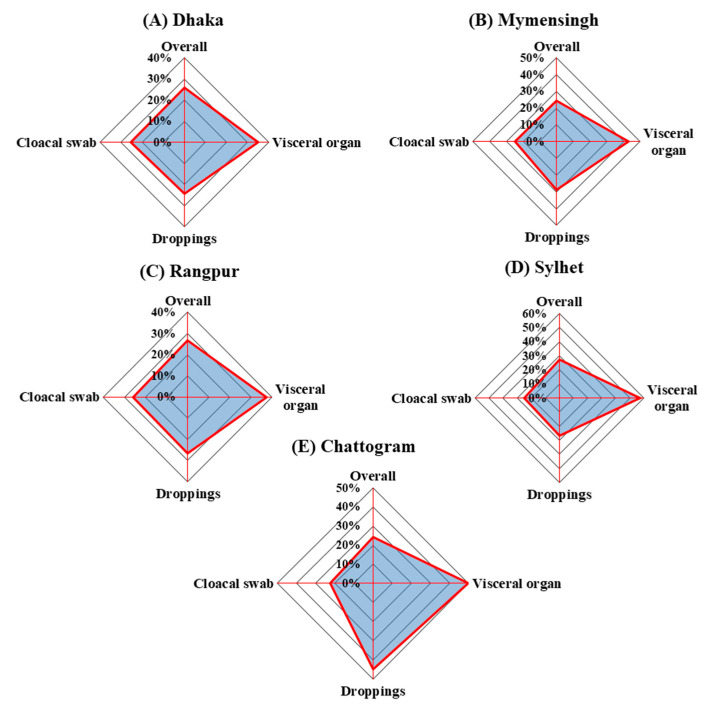
Occurrence of *Salmonella* Gallinarum in different samples (cloacal swab (*n* = 535), visceral organ (*n* = 50) and drooping (*n* = 180)) collected from 12 layer flocks of five divisions: (**A**) Dhaka, (**B**) Mymensingh, (**C**) Rangpur, (**D**) Sylhet, and (**E**) Chattogram. Overall occurrence of *Salmonella* Gallinarum was estimated as 25.9%, 24.6%, 26.8%, 27.4%, and 24.4% in Dhaka, Mymensingh, Rangpur, Sylhet, and Chattogram divisions, respectively.

Occurrence of *Salmonella* Gallinarum was detected in 25.90 (57/220)%, 24.60 (41/61)%, 26.80(37/138)%, 27.40(32/117)%, and 24.40(30/123)% samples collected from Dhaka, Mymensingh, Rangpur, Sylhet, and Chattogram divisions, respectively, with an average occurrence of 25.8% (95% CI: 22.7–29). The observed variations in division-wise occurrence of the bacterium was found to be statistically non-significant (p = 0.97) (Figure 2). One the other hand, of the 12 flocks surveyed in this study, 75% (9/12) were found to be positive for *Salmonella* Gallinarum (Supplementary Table S1). However, the occurrence of *Salmonella* Gallinarum in different samples (cloacal swab, droppings, and visceral organ) at individual farms showed more wide-ranging variations, i.e., from 0% to 56.9% of total samples (Figure 2).

### 3.2. Sequencing and Phylogenetic Analysis

**Figure 3:**
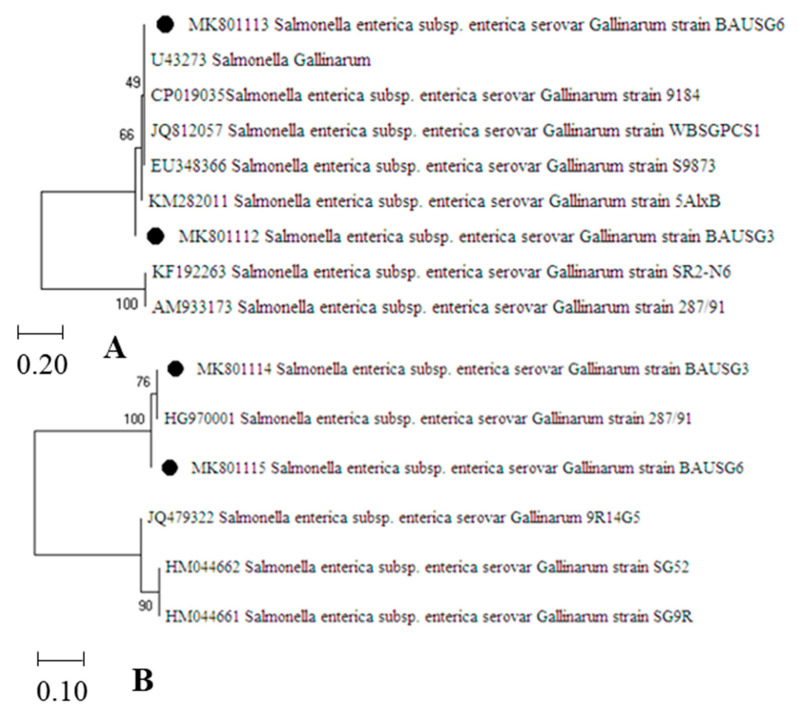
Phylogenetic analysis of (**A**) invasive gene (*inv*A) and (**B**) plasmid virulence gene (*spv*C) of *Salmonella* Gallinarum strains (BAUSG3 and BAUSG6). The phylogenetic tree was constructed using the Neighbour-Joining (NJ) method with comparative alignment of 266 nucleotides of *invA*; and 572 nucleotides of *spvC*. BAUSG3 and BAUSG6 are marked as black circles in the phylogenetic tree, comparing their affinity to selected sequences of the target genes downloaded from the GenBank.

Gene *(inv*A *and spv*C*)* based PCR assays, followed by sequencing and phylogenetic analysis of the representative target genes confirmed the identity of *Salmonella* Gallinarum strains. BLAST analysis confirmed a 100% similarity of *invA* gene between the isolated strains of *Salmonella* Gallinarum, namely BAUSG3 and BAUSG6. These nucleotide sequences of *invA* gene showed a close homology between 99.21% and 98.05% to earlier reported strains isolated from India (Accession number: JQ812057.1), China (EU348366.1), Korea (KF192263.1), Egypt (KM282011.1), UK(AM933173.1), and the USA (CP019035.1) (Figure 3A). Phylogenetic analysis of *inv*A genes revealed the representative strains clustering to a lineage, which comprised of five previously published sequences of *Salmonella* Gallinarum strains from different countries. Similarly, nucleotide sequences of the *spv*C genes of *Salmonella* Gallinarum strains (BAUSG3 and BAUSG6) were found to be closely related with the previously published sequences of *spvC* of the same serogroup strains isolated from Korea and the UK (Figure 3B). According to BLAST analysis, a 97.8% similarity with five mismatches between the nucleotide sequences of the *spv*C genes was observed for the isolated strains, BAUSG3 and BAUSG6. However, these *spv*C genes showed between 99.82% and 97.51% homology to previously published sequences of this gene in strains isolated from different countries. The nucleotide sequences generated in this study were submitted in the GenBank and are available under accession numbers: MK801112 and MK801113 (*inv*A1 and *inv*A2, respectively) and MK801114 and MK801115 *(spv*C1 and *spv*C2, respectively).

### 3.3. Antibiogram

#### 3.3.1. Antimicrobial Susceptibility Status

**Figure 4:**
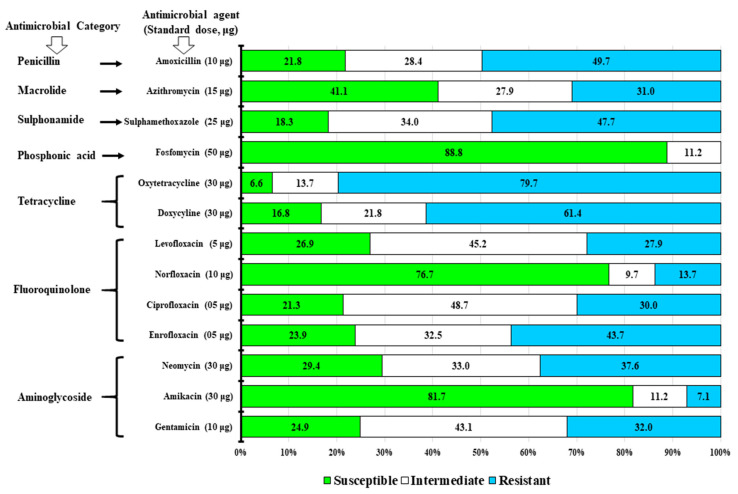
Antimicrobial susceptibility test results of *Salmonella* Gallinarum (*n* = 197) isolated from the non-vaccinated layer flocks. A total of 13 antimicrobial agents under seven groups of antimicrobials were used at standard doses (μg): Amoxycillin (10 µg), Azithromycin (15 µg), Sulfamethoxazole (25 µg), Fosfomycin (50 µg), Oxytetracycline (30 µg), Doxycycline (25 µg), Levofloxacin (5 µg), Norfloxacin (10 µg), Ciprofloxacin (5 µg), Enrofloxacin (5µg), Neomycin (30 µg), Amikacin (30 µg), and Gentamicin (10 μg). Three categories (susceptible, intermediate, and resistant) of the resistant pattern in accordance to Clinical and Laboratory Standards Institute (CLSI) [42] were estimated.

Among 197 isolated strains of *Salmonella* Gallinarum, confirmed by the PCR assays, 88.8%, 81.7%, and 76.7% were found to be susceptible to Fosfomycin, Amikacin, and Norfloxacin, respectively, while 48.7%, 45.2%, and 43.1% isolates could be considered as intermediately resistant to Ciprofloxacin, Levofloxacin, and Gentamicin, respectively. On the other hand, 79.7%, 61.4%, 49.7%, 47.7%, 43.7%, and 37.6% isolates of this serogroup were found to be fully resistant to Oxytetracycline, Doxycycline, Amoxycillin, Sulfamethoxazole, Enrofloxacin, and Neomycin, respectively (Figure 4).

#### 3.3.2. Antimicrobial Resistance Pattern

Among the isolated strains (*n* = 197) of *Salmonella* Gallinarum, 19.9% (*n* = 39) were observed to be resistant to one of the antimicrobial agents (OT, SXT, AMX, DO), whereas 13.7% (*n* = 27) showed resistance against two antimicrobials (OT-SXT and OT-AMX). Notably, a high prevalence of multidrug resistance (MDR), comprising 66.5% (131/197) of isolated strains, was observed. The MDR strains of *Salmonella* Gallinarum showed diverse patterns considering their resistance traits against three to six antimicrobial agents. Among these MDR strains, the observed resistance traits against three and four antimicrobials could be differentiated into two and one pattern(s), namely, OT-CN-AMX (11.7%, *n* = 23) and CIP-DO-AZM (9.6%, *n* = 19), and ENR-OT-SXT-AMX (8.1%, *n* = 16), respectively. Similarly, the MDR traits characterized by resistance to five antimicrobials were found to comprise of two patterns, N-DO-SXT-AZM-AMX (9.6%, *n*=19) and GEN-LEV-OT-SXT-AMX (8.1%, *n* = 16). The MDR strains showing resistance against six antimicrobials, could be also differentiated into two patterns: N-LEV-AZM-OT-SXT-AMX (10.7%, *n* = 21) and CN-N-ENR-DO-SXT-AMX (8.6%, *n* = 17) (Table 6).

## 4. Discussion

Salmonellosis is considered as one of the major causes of decreased meat and egg production in commercial poultry. The occurrence of disease has been reported to be connected with inadequate biosecurity measures in poultry farming practices in Bangladesh [20,44]. It has been difficult to evaluate the actual disease burden and adopt appropriate interventions in small-scale commercial poultry farms of Bangladesh due to limited systematic information on decreased egg production in layer flocks associated with salmonellosis, particularly, *Salmonella* Gallinarum infection. Of the surveyed farms scattered over different districts in five divisions in Bangladesh, a huge majority (75%, 9/12 farms) of the layer flocks were found infected with *Salmonella* Gallinarum, which could be connected to decreased egg production [18,29,45]. Considering its significance, *Salmonella* Gallinarum infection in the poultry sector has been included within the important notifiable diseases of the World Organization for Animal Health (OIE), whereas there has been an increased attention to implement a strict control measures regarding the import of birds and eggs [46].

Observations made in this study showed the occurrence of *Salmonella* spp., detected by biochemical tests and genus-specific 16S rRNA assay, in 26.8% (205/765, 95% CI: 23.7–30.1) samples of layer flocks. Interestingly, an overwhelming occurrence in these samples, 25.8% (197/765, 95% CI: 22.7–29), of *Salmonella* Gallinarum, confirmed by differential biochemical tests and molecular assays targeting virulence genes, *inv*A and *spv*C was revealed. The observed large-scale contamination in visceral organs of *Salmonella* Gallinarum can be related to the typical invasive feature of Fowl Typhoid (FT), which causes lesions in multiple organs, including liver, heart, spleen, ovary, and intestine. In congruence, a higher prevalence of this virulent serogroup in visceral organs (42%, 21/50) in comparison to dropping (26.1%, 47/180) and cloacal swabs (24.1%, 129/535) was notable. A ubiquitous occurrence of *inv*A and *spv*C genes observed for the isolated strains (*n* = 197) of *Salmonella* Gallinarum is in congruence to their prevalence reported previously among the virulent serotypes of *S. enterica* [10]. The observed similarity in the sequences of virulence *inv*A and *spv*C genes of *Salmonella* Gallinarum strains obtained in this study indicates their genetic relatedness to the *Salmonella* Gallinarum strains isolated from poultry sources in different counties, including India, China, Korea, Egypt, UK, and the USA. Sequencing of *inv*A and *spv*C in a higher number of *Salmonella* Gallinarum strains would enrich our knowledge to better understand the evolutionary trend linked to the geospatial prevalence of these virulent genes in *Salmonella* spp.

The overall occurrence of *Salmonella* Gallinarum in 25.8% (197/765) of the samples is lower than a previously reported occurrence in 53.5% of the samples of layer flocks in Bangladesh [21]. However, as observed in the present study, this pathogenic serovar can contaminate the majority samples (at least 57%) of the poultry flocks in an individual farm. On the other hand, a variable occurrence, between 11.5% and 24%, of *Salmonella* spp. reported for poultry samples in different geographical locations, e.g., France, Japan, Tanzania, and United Kingdom, respectively [47,48,49,50], could be attributable to geo-climatic variations. Nonetheless, differential anthropogenic risk factors, namely, age of the birds, flock size, feed, hygienic condition of the farm, and environmental determinants, including transmission from poultry litter, pest, and rodent may influence the preponderance of pathogenic microbes such as *Salmonella* Gallinarum in small-scale poultry farms [51,52].

Among the potential biosecurity interventions, systematic vaccination programs based on surveillance studies on the pathogenic microbes circulating in a particular region is a key approach to effectively control the infection of virulent strains, including *Salmonella* serotypes, in poultry flocks [53]. The poultry farms surveyed in this study were selected based on their non-vaccinated status, imposing a higher likelihood of diseases from *Salmonella* Gallinarum in reared layer flocks [54]. At present, a few imported commercial vaccines are available for immunization of layer poultry flocks in Bangladesh. Notably the SG 9R strain of *Salmonella* Gallinarum is being used as FT vaccine (viz. Nobilis^®^ SG 9R, MSD Animal Health). However, this vaccine may not be antigenically well-matched with the circulating strains. Phylogenetic analysis of the nucleotide sequences of a virulence gene, *spv*C, obtained from a couple of *Salmonella* Gallinarum strains of this study, displayed its close relatedness to that of the SG 9R strain (accession number: HM044661). This kind of locally adapted strain could be considered as a more potential candidate strain to develop antigenically well-matched vaccine for *Salmonella* Gallinarum strains circulating in Bangladesh.

The rise of MDR among *Salmonella* spp. is a growing concern worldwide, particularly in developing countries, where multiple antibiotics are indiscriminately used at poultry farms for enhanced production [19,55,56,57]. The present study showed that 48.7%, 45.2%, and 43.1% isolates were intermediately resistant to Ciprofloxacin, Levofloxacin, and Gentamcin, respectively, however, 79.7%, 61.4%, 49.7%, 47.7%, 43.7%, and 37.6% isolates were fully resistant to Oxytetracycline, Doxycycline, Amoxycillin, Sulfamethoxazole, Enrofloxacin, and Neomycin. These findings are in congruence to the results of earlier studies [25,57,58,59]. Bangladesh is already included among the countries at high risk of AMR, according to the WHO [60]. Likewise, this study reporting 66.5% (*n* = 131) *Salmonella* Gallinarum isolates as resistant to three to six antimicrobials, indicates an alarming consequence to the extensive use of different antibiotic classes in layer flocks.

The low biosecurity standards in majority (>60%) of the small-scale commercial poultry farms, together with no vaccination status, and unscrupulous use of antibiotics might have substantiated the high occurrence of MDR strains of *Salmonella* Gallinarum in the layer flocks in Bangladesh [51,61]. Apart from further infections caused by this kind of MDR *Salmonella* Gallinarum strains, reportedly also resistant to prophylactic antibiotics [62], exposure of residual antimicrobials through the food chain is considered to impose a significant hazard to public health [63]. Therefore, training programs to enrich farmers’ knowledge on Good Agriculture Practices (GAP) including prudent use of antibiotics, immunization of layer birds and other biosecurity measures, including hygiene, water and waste management [24,64,65], would be vital to lessen the *Salmonella* Gallinarum infection in poultry farms.

The limited sampling scheme employed for each of the study farms did not capture any temporal variation in the occurrence of *Salmonella* Gallinaruam in layer flocks. Because of high phenotypic similarity between the closely related serovars, *Salmonella* Gallinarum and *Salmonella* Pulloram, a number of biochemical tests and serotyping were required to substantiate molecular detection targeting a couple of genes, including their sequencing and phylogenetic interpretation, of *Salmonella* Gallinarum isolates under this study. Culture-based biochemical identification methods have some inherent drawbacks, e.g., being labor-intensive and time consuming (requiring five to seven days). Another limitation of the methodological approach was the qualitative estimation of the bacterial occurrence, whereas a quantitative approach could capture more detail of the variations. On the other hand, sequencing and phylogenetic analysis of the target genes were done for only a few isolates. These could be considered as the primary limitations or constrains of this study.

## 5. Conclusions

The present study clearly shows a widespread occurrence of *Salmonella* Gallinarum strains with MDR traits in small-scale commercial layer flocks in all the major divisions of Bangladesh. The updated information on AMR patterns of *Salmonella* spp. circulating in the poultry flocks will contribute in efforts to lessen the multi-spectrum hazards, including treatment failure and production loss associated with the large-scale infection of MDR *Salmonella* Gallinarum in the poultry sector. Results obtained from the surveyed farms indicate an indispensable need of promoting biosecurity measures, and farmers’ training on GAP, including strict vaccination, and prudent use of antimicrobials. This study will eventually benefit the policy makers in divulging a strategic framework for the improvement of farmers’ livelihood, health and food security and safety in the context of the alarming MDR infections in poultry flocks at low resource settings.

## Figures and Tables

**Table 1:** List of primers used in this study.

Gene	Sequence (5′-3′)	Amplicon Size (bp)	Annealing Temperature	Reference
Sal 16S rRNA F	TGTTGTGGTTAATAACCGCA	574	50 °C	[40]
Sal 16S rRNA R	CACAAATCCATCTCTGGA			
*inv*A-S139	GTG AAATTATCGCCACGTTCGGGCAA	284	64 °C	[17]
*inv*A-S141	TCATCGCACCGTCAAAGGAACC			
*spv*C-SPV1	ACTCCTTGCACAACCAAATGCGGA	571	64 °C	[10]
*spv*C-SPV2	TGTCTTCTGCATTTCGCCACCATCA			

**Table 2:** Occurrence of *Salmonella* isolates, determined by selective culture and biochemical tests, in different samples collected from 12 layer flocks from 5 divisions of Bangladesh.

Method/Type of Sample	Number of Isolates	Occurrence (with 95% Confidence Interval)	*p*-Value (Pearson’s Chi-Squared Test)
Selective culture (*Salmonella* spp.)			
Cloacal swab (*n* = 535)	138	25.8 (22.1–29.7)	0.003
Visceral organ (*n* = 50)	24	48 (33.7–62.6)	
Droppings (*n* = 180)	52	29 (22.4–36.1)	
Total sample (*N* = 765)	214	28 (24.8–31.3)	
Biochemical identification (*Salmonellla* Gallinarum)			
Cloacal swab (*n* = 138)	130	24.3 (20.7–28.2)	0.02
Visceral organ (*n* = 24)	21	42 (28.2–56.8)	
Droppings (*n* = 52)	48	26.7 (20.4–33.8)	
Total sample (*N* = 214)	199	26 (22.9–29.3)	

**Table 3:** Results of different biochemical test among culture positive isolates of *Salmonella* spp. (*n* = 214).

Biochemical Tests	*Salmonella* Gallinarum	*Salmonella* Typhimurium	*Salmonella* Pullorum	Others
Carbohydrate fermentation				
Glucose	+	+	+	-
	(Acid)	(Acid and Gas)	(Acid and Gas)	-
Dulcitol	+	+	-	-
	(Acid)	(Acid)		
Maltose	+	+	±	-
	(Acid)	(Acid and Gas)	(Acid and Gas)	
Indole production	-	-	-	-
Methyl red test	+	+	+	-
Voges-Proskauer test	-	-	-	-
Motility	-	+	-	-
Total isolates	199	6	0	9

+: Positive; -: Negative.

**Table 4:** Occurrence of *Salmonella* isolates in different samples collected from 12 layer flocks from five divisions of Bangladesh as confirmed though molecular assays.

Type of Sample	Number of Isolates	Occurrence (with 95% CI *)	*p*-Value (Pearson’s Chi-Squared Test)
16S rRNA gene based PCR (*Salmonella* spp.)			
Cloacal swab (*n* = 535)	134	25 (21.4–28.9)	0.015
Visceral organ (*n* = 50)	22	44 (30–58.7)	
Droppings (*n* = 180)	49	27.2 (20.9–34.3)	
Total sample (*N* = 65)	205	26.8 (23.7–30.1)	
*inv*A and *spv*C gene based PCR (*Salmonella* Gallinarum)			
Cloacal swab (*n* = 535)	129	24.1 (20.5–28)	0.02
Visceral organ (*n* = 50)	21	42.0 (28.2–56.8)	
Droppings (*n* = 180)	47	26.1 (20–33.2)	
Total sample (*N* = 765)	197	25.8 (22.7–29)	

* CI: Confidence Interval.

**Table 5:** Results of serogrouping in *Salmonella* positive isolates (*n* = 205).

Isolate (*n*)	No. of Serogroup (%)			
	Poly A-I	Group B (O: 4, 5, 27)	Group C (O: 6, 7, 8, 14, 20)	Group D (O: 9, 46)
*Salmonella* spp. (205)	205 (100)	4 (2%)	2 (1%)	199 (97%)

O: Somatic antigen of *Salmonella.*

**Table 6:** Antimicrobial resistance patterns of *Salmonella* Gallinarum isolated from the layer flocks.

Resistance Against Antimicrobials	Resistance Patterns	*Salmonella* Gallinarum Isolates (*n* = 197)	
		No. (%) of Strains	Subtotal (No. (%))
Against one to two antimicrobial agents			
Against one	OT	15 (7.6)	39 (19.9)
	SXT	9 (4.6)	
	AMX	7 (3.6)	
	DO	8 (4.1)	
Against two	OT, SXT	9 (4.6)	27 (13.7)
	OT, AMX	18 (9.1)	
Against three or more antimicrobial agents (multidrug resistance)			
Against three	CIP, DO, AZM	19 (9.6)	42 (21.3)
	OT, CN, AMX	23 (11.7)	
Against four	ENR, OT, SXT, AMX	16 (8.1)	16 (8.1)
Against five	N, DO, SXT, AZM, AMX	19 (9.6)	35 (17.7)
	GEN, LEV, OT, SXT, AMX	16 (8.1)	
Against six	CN, N, ENR, DO, SXT, AMX	17 (8.6)	38 (19.3)
	N, LEV, AZM, OT, SXT, AMX	21 (10.7)	
Against three or more antimicrobials		131 (66.5)	

N: Neomycin; GEN: Gentamicin; ENR: Enrofloxacin; CIP: Ciprofloxacin; LEV: Levofloxacin; DO: Doxycycline; OT: Oxytetracycline; SXT: Sulfamethoxazole; AZM: Azithromycin, and AMX: Amoxicillin.

## Data Availability

All data relevant to this study are included in this manuscript and Supplementary Materials.

## Supplementary Materials

The data that support the findings of this study are available online at https://www.mdpi.com/article/10.3390/vetsci8050071/s1. Table S1: Description of the surveyed farms with type of sample collection and farm level positivity status (*N* = 12), and Supplementary Table S2: Sample collection checklist.
