# Supplementary material for: Salmonella Gallinarum in Small-Scale Commercial Layer Flocks: Occurrence, Molecular Diversity and Antibiogram
Source: Vet Sci. 2021 Apr 23;8(5):71. doi: 10.3390/vetsci8050071 (PMC8145292; doi:10.3390/vetsci8050071)
Supplement: Supplementary file 1 [file vetsci-08-00071-s001.zip › Supplementary Table S1.pdf]

Supplementary Table S1: Description diversity of the surveyed farms, with type of sample collection and farm-wise variation in the occurrence of *S.* Gallinarum farm level positivity status (N=12)

**Division (region)**	**District**	**Flock ID**	**Flock Size**	**Breed**	**Age of production fall (wks)**	**No. of samples collected**			**Total**	**PCR positives isolates (Specific for *inv*A and *spv*C genes)**			**Total**	**Farm level occurrence (%)**
						**CS**	**VO**	**D**		**CS**	**VO**	**D**		
Dhaka (central)	Gazipur	D3	≤1500	Hy-Line Brown	39	50	8	15	73	15	2	3	20	27.39
	Gazipur	D5	≤2500	Shaver Brown 579	45	60	12	15	87	13	5	3	21	24.13
	Tangail	D6	≤1100	Shaver Brown 579	47	45	0	15	60	11	0	5	16	26.67
Mymensingh (Northern)	Mymensingh	N8	≤1300	Hy-Line Brown	51	30	0	15	45	0	0	0	0	-
	Jamalpur	D9	≤1100	ISA Brown	44	35	0	15	50	0	0	0	0	-
	Netrkona	D20	≤1400	Hisex Brown	38	50	7	15	72	25	3	13	41	56.94
Rangpur (North west)	Dinajpur	L15	≤1500	Hy-line Brown	42	45	0	15	60	13	0	5	18	30.00
	Bogura	L20	≤2000	ISA Brown	48	55	8	15	78	13	3	3	19	24.36
Sylhet (North east)	Habiganj	P2	≤1000	Shaver Brown 579	43	35	0	15	50	0	0	0	0	-
	Maulvibazar	K9	≤1500	ISA Brown	39	45	7	15	67	20	4	8	32	47.76
Chottogram (Southern)	Chottogram	M1	≤2000	Hisex Brown	51	50	0	15	65	11	0	2	13	20.00
	Feni	S8	≤1000	Hy-Line Brown	38	35	8	15	58	8	4	5	17	29.31
**Overall**						**535**	**50**	**180**	**765**	**129**	**21**	**47**	**197**	**25.75**

Note: CS= Cloacal Swab, VO= Visceral Organs, D=Droppings
