# Supplementary material for: Salmonella Gallinarum in Small-Scale Commercial Layer Flocks: Occurrence, Molecular Diversity and Antibiogram
Source: Vet Sci. 2021 Apr 23;8(5):71. doi: 10.3390/vetsci8050071 (PMC8145292; doi:10.3390/vetsci8050071)
Supplement: Supplementary file 1 [file vetsci-08-00071-s001.zip › Supplementary Table S2.pdf]

**Supplementary Table S2:** Sample collection checklist

**Name of the project:** *Salmonella* Gallinarum in Small-scale Commercial Layer Flocks: Occurrance, Molecular Diversity and Antibiogram

**Sample collection checklist code # ……..**

**Flock Details:**

Layer Flock code: …………

Flock size : …………

Age (week's) : …………

**Geo-spatial location:**

Northing…………………… Easting………………………

Location of layer farm:

Village:………………….., Sub-district (Upazila): ………. , District: …………

**Breed details:**

Source of Layer DOC (Breed): ………..

Source of Feed (Self made / private feed mill company): ……………………….

**Sampling time:**

Expected time of drop of egg production: ………………………….

Sampling also done during any abnormality observed in egg production: …………..

Any medicine/ chemicals were given to the flock while sampling?: Y / N

If yes: Please mention the name of medicine with dose: ……………………..

**Sample Code #**

| **Sample type** | **Sample ID** | **Remarks** |
|---|---|---|
| Cloacal swab | | |
| Droppings | | |
| Whole carcass | | |

**Signature ……………………**
**(Research assistant/fellow)**

**Signature ………………………………..**
**(Research Supervisor)**
